# Self-Assessment of Adherence to Medication: A Case Study in Campania Region Community-Dwelling Population

**DOI:** 10.1155/2015/682503

**Published:** 2015-08-05

**Authors:** Enrica Menditto, Francesca Guerriero, Valentina Orlando, Catherine Crola, Carolina Di Somma, Maddalena Illario, Donald E. Morisky, Annamaria Colao

**Affiliations:** ^1^Center of Pharmacoeconomics (CIRFF), Federico II University, 80131 Naples, Italy; ^2^Research and Development Board, Federico II University Hospital, 80131 Naples, Italy; ^3^Translational Medical Sciences (DISMET), Federico II University, 80131 Naples, Italy; ^4^IRCCS SDN, 80131 Naples, Italy; ^5^Department of Community Health Sciences, UCLA Fielding School of Public Health, Los Angeles, CA 90095-1772, USA; ^6^Department of Clinical Medicine and Surgery, Federico II University, 80131 Naples, Italy

## Abstract

*Objectives*. The aim of the study was to assess self-reported medication adherence measure in patients selected during a health education and health promotion focused event held in the Campania region. The study also assessed sociodemographic determinants of adherence. *Methods*. An interviewer assisted survey was conducted to assess adherence using the Italian version of the 8-item Morisky Medication Adherence Scale (MMAS-8). Participants older than 18 years were interviewed by pharmacists while waiting for free-medical checkup. *Results*. A total of 312 participants were interviewed during the Health Campus event. A total of 187 (59.9%) had low adherence to medications. Pearson's bivariate correlation showed positive association between the MMAS-8 score and gender, educational level and smoking (*P* < 0.05). A multivariable analysis showed that the level of education and smoking were independent predictors of adherence. Individuals with an average level of education (odds ratio (OR), 2.21, 95% confidence interval (CI), 1.08–4.52) and nonsmoker (odds ratio (OR) 1.87, 95% confidence interval (CI), 1.04–3.35) were found to be more adherent to medication than those with a lower level of education and smoking. *Conclusion*. The analysis showed very low prescription adherence levels in the interviewed population. The level of education was a relevant predictor associated with that result.

## 1. Introduction

Medication adherence is a growing concern to healthcare systems as nonadherence to pharmacotherapy has been associated with adverse outcomes and higher costs of care.

Medication nonadherence is likely to grow as the population ages and as patients need to take more medications to treat chronic conditions. Several studies highlighted that the levels of adherence to treatment in patients with chronic diseases are inadequate showing rates that do not exceed 50%. Adherence and persistence to drug treatment represent the key factors necessary to gain a significant reduction in morbidity and mortality and to optimize the use of financial resources, but these aspects are widely underestimated in clinical practice and by patients [1–5]. The World Health Organization (WHO) estimates that the cost of nonadherence to drug therapy amounts to 125 million euros per year in Europe [6].

The approaches used to assess medication adherence include direct or indirect methods. Indirect methods include patient interviews, pill counts, refill records, and measurement of health outcomes. However, the most practical approach to apply in clinical practice is patient self-report. The advantages of assessing medication adherence by self-report included simplicity, speed, and viability of use [7]. The Morisky Medication Adherence Scale (MMAS-8) is one of the most commonly used self-reporting methods [8, 9].

In addition, the MMAS-8 provides information on behaviors associated with low adherence that may be unintentional (e.g., forgetfulness) or intentional (e.g., stopped taking medication(s) without telling the doctor, because they felt worse when they took it). Identification of these behaviors can facilitate tailoring of interventions to specific patient issues and is strongly related to concordance [7, 10].

Currently there are many studies evaluating the degree of adherence to treatment in patients with specific diseases [7, 11–13], while there seems to be a lack in the literature related to adherence to treatment in the general population.

The purpose of the current analysis was to evaluate adherence to medication in the general population during a prevention-related event held in Campania region (Southern Italy). The interview was carried out by administering the Italian version MMAS-8 questionnaire.

## 2. Materials and Methods

### 2.1. Setting

The prevention-related event was organized by the Health Campus, a nonprofit organization. It was established to carry out a continuous activity of dissemination and promotion of prevention through specific clinical screening and educational initiatives. The study is part of a larger activity that started in 2010 to assess the health status of the general population of the Campania Region, by providing free consultation, visits, and diagnostics for people coming to the outdoor hospital held in different public squares of Campania region during popular events [14].

The Health Campus focuses its energy and resources primarily on two major objectives: first, to provide specialist visits for disease prevention and early detection of risks to the health of Campania citizens; second, to promote dissemination of a culture of prevention that encourages a change in lifestyle for healthy living.

The Health Campus events periodically set up a disease prevention “Field Clinic,” where early diagnosis tests are offered to the general public free of charge. Thus the public is encouraged to undergo specialist examinations and is informed about health risks and good practice habits to adopt for early diagnosis and screening.

The Health Campus is typically set up during large, popular events or at the request of public and/or private organizations.

### 2.2. Study Design

A cross sectional assisted interview survey was conducted by pharmacists on a population group selected during the Health Campus event held in Naples in April 2013 during the America's Cup.

People who were attending the event and were interested to undergo clinical evaluation and/or to take the opportunity of the available screening services, upon arrival, were selected as eligible if they were 18 years or older and taking at least one medication for at least one chronic condition. While waiting to receive a medical checkup, they were invited to the assessment of medication adherence and were interviewed by pharmacists. All participants provided their written informed consent to the study that was conducted in line with the Helsinki declaration for human studies.

The information collected included demographic data (i.e., age and gender), level of education (referred to Italian education system), smoking habit, number of drugs taken, and type of chronic disease present such as hypertension, diabetes, heart failure, COPD, renal failure, and osteoporosis. Self-reported adherence was assessed using the Italian validated version of the Morisky Medication Adherence Scale (MMAS-8) [15]. Use of the MMAS is protected by US copyright laws. Permission for use is required. This scale has been validated and used in many languages [12, 15, 16]. It consists of eight items that address specific behavior regarding medication assumption and both intentional and unintentional adherence (questionnaire used for interview is reported as Supplementary Material available online at http://dx.doi.org/10.1155/2015/682503, Table S1).

Scores on the Morisky Medication Adherence Scale can range from 0 to 8, scores < 6 reflecting low adherence, scores ≥ 6 < 8 reflecting medium adherence, and scores = 8 reflecting high adherence [17].

In this study, none of the interviewed participants reported high adherence on the Morisky scale, so the dependent variable of patient adherence was dichotomized into 2 groups: low adherence (scores < 6) versus medium adherence (scores ≥ 6) [18].

The MMAS provides information on behaviors associated with low adherence that may be unintentional (e.g., forgetfulness) or intentional (e.g., stopped taking medication(s) without telling the doctor, because they felt worse when they took it) [7]. The MMAS-8 is divided into 4 items that assess intentional nonadherence and 4 items that assess unintentional nonadherence. We divided participants with low adherence into two categories: intentional nonadherent when majority of answers provided indicate a behavior that is not related to forgetfulness and unintentional nonadherent when majority of answers provided indicate a behavior that is related to forgetfulness.

### 2.3. Statistical Analyses

Baseline characteristics of the study population were analyzed using descriptive statistics and their degree of adherence, chi square test, and Student's* t*-test were used where appropriate. Pearson's correlation was used to assess bivariate association between adherence score and participant characteristic.

Additionally, associations between adherence and covariates were evaluated after accounting for other variables and predictors using logistic regression. The initial model included gender, smoking, and educational level. Factors were then sequentially eliminated from the multivariate model until only factors significant at the level of* P* < 0.05 remained in the final model. All analyses were performed using SPSS software version 17.1 for Windows (SPSS Inc., Chicago, IL, USA).

## 3. Results

312 patients were interviewed during the Health Campus event. The mean age was 61.8 (SD ±11.1) years. 57.4% of participants were women.

The characteristics of the analyzed population and the results of univariate association between low adherence on the Morisky scale and patient-reported factors are reported in Table 1.

18.9% of patients have a degree graduation, 35.3% of patients had high school graduation whereas 29.8% of patients attended secondary school graduation, and 16.0% finished the primary schools.

A total of 40.1% of patients had 2 or more diseases. More specifically, patients with hypertension were 169 (54.2%); 37 (11.9%) had type II diabetes; 29 (9.3%) had heart failure; 26 (8.3%) had osteoporosis; 22 (7.1%) had gastritis; 5 (1.6%) had COPD; and 4 (1.3%) had renal failure. A total of 37.5% of patients took from 2 to 3 drugs and 59.9% of patients have at least one disease. A total of 76.9% of patients were nonsmoking.

In the overall population, 59.9% of participants demonstrated low adherence and 40.1% medium adherence. Nobody showed high adherence. The mean score for the medication adherence scale was 4.95 (±1.52). Table 1 shows the characteristics of the population and the significant results (*P* < 0.05) of the univariate analyses examining the associations between scores on the Morisky scale and patient-reported factors.

Considering the frequency distribution of the MMAS-8 items, 42.6% of patients answered that they forgot to take their medicines, 47.1% of patients had problems taking their medicines in the last two weeks, 21.8% of patients have stopped taking their medicines without telling their doctor, 55.4% of patients stated that when they travel they forgot to bring along their medication, 20.2% of patients stated that when they feel like their condition is under control, they stop taking their medication, and 18.3% of patients felt hassled about sticking to their treatment plan. In conclusion, 19.9% of patients have difficulty remembering to take all their medications.

A total of 60.9% of nonadherent participants were intentional, 13.4% of nonadherent subjects were unintentional, and 25.7% of nonadherent participants did not fall within either category.

The number of medications prescribed, age, and number of diseases are not significant.

However, educational level, smoking habit, and gender were statistically significant (*P* = 0.01; 0.03; 0.03, resp.) (Table 1).

Univariate analysis showed that males have a higher risk of being low adherent than women (odds ratio (OR) 1.67, 95% confidence interval (CI), 1.05–2.66); the risk of being nonadherent to treatment for smokers is about double compared to nonsmokers (odds ratio (OR) 0.54, 95% confidence interval (CI) 0.30–0.95).

Only 2 factors remained as significant independent predictors of high adherence after logistic regression adjustment: level of education (*P *= 0.03) and smoking habit (*P* = 0.03) (Table 2).

People with high school graduation (odds ratio (OR), 2.21, 95% confidence interval (CI), 1.08–4.52) and nonsmokers (odds ratio (OR) 1.87, 95% confidence interval (CI), 1.04–3.35) are more adherent to treatment than those with lower education and smokers (Table 2).

## 4. Discussion

To the best of our knowledge, this is the first survey about adherence to medication that was carried out during a prevention event, among a population selected at the community level.

Previous studies on adherence have mostly been restricted to specific clinical populations, a specific disease, and/or a single treatment and the relevance of the outcomes to prescription drug users in general has been unclear [19]. Patient compliance to drug therapy is one of the most relevant issues in clinical practice as the success of a therapeutic intervention depends on the actual patient adherence to therapy. The noncompliance to treatment has direct consequences such as the distortion of the effectiveness of the treatment [20]. Furthermore, several observational studies raised many concerns about undertreatment in current clinical practice, because it leads to an increase in the number of hospitalizations and consequently an increase of the cost for the health system [21].

In industrialized countries, adherence to treatment by patients with chronic diseases is about 50% [22]. Our results are in line, showing 59.9% of low adherence to medications.

The main causes of poor adherence are the lack of motivation of the patients as well as low average education level, limited awareness about health, and the lack of perception of risk arising from treatment discontinuation [11]. This is in line with our results that showed that low level of education is a predictor of low adherence.

Moreover different studies indicate that 30% of the interviewed participants claim forgetfulness as the reason for their nonadherence, thus making it a prominent reason for nonadherence [23].

Using the MMAS-8, we were able to identify intentional and unintentional behaviors that cause low adherence [7]. About 42.6% of respondents say they do not take their medication regularly due to forgetfulness and 21.8% stop their treatment without speaking to their doctor. Intentional nonadherence indicates a lack of understanding and misconceptions regarding the chronicity of a health condition. This behavior can be related to lack of concordance, intended as the fact that the “prescriber and patient should come to an agreement about the regimen that the patient will take” [10]. Taking medications continuously and according to the instructions of the physician is an important aspect of drug treatment. This aspect, however, does not seem to be considered by the patients: our analysis showed that no more than 40.1% of patients have a medium level of adherence.

The interview was carried out by pharmacists. Several studies showed that interventions by pharmacists, often as a part of the care team, jointly with clinicians, have been found to be effective in improving medication adherence [24, 25]. However in Italy pharmacists are not sufficiently involved in adherence-related activities, despite the fact that pharmacists are the last point of contact with the patient before beginning the use of the medications [26]. Strategies should be formulated to achieve greater involvement of pharmacists in promoting adherence [27]. For this reason we chose to involve the pharmacist in the administration of the questionnaire as a way to improve awareness about adherence-related issues among pharmacists as well.

The European Innovation Partnership A1 Action Group on Active and Healthy Ageing, in 2012, has implemented joint initiatives between various European countries to improve the quality of the prescription and adherence to treatment [28]. Many intervention strategies that are currently being discussed among partners are aimed to promote coordinated and multidisciplinary intervention by involving all major stakeholders (general practitioners and specialists, pharmacists, nurses, family, health authorities, and pharmaceutical industry). The present initiative is part of the strategy carried out by the EIP-AHA A1 Action Group and provides preliminary data that might be useful for the further focused interventions.

### 4.1. Limitations of the Study

This study has some obvious limitations. Participation in this study was voluntary and, as a result, there may have been a selection bias. Population has not been selected on the basis of specific diseases or according to the number of medications prescribed. Nevertheless we assume that to be a minor limitation as the study was not aimed to assess adherence in specific conditions. Our results give a first overview about level of adherence and predictive factors. A larger population could provide more robust results. The assessment of adherence in our study was based on self-reporting. Questionnaires are a self-reporting tool that is relatively simple and economical to use, but it could overestimate the rate of medication adherence [29, 30].

## 5. Conclusion

In conclusion, we provided information about adherence levels in a population selected among participants to a prevention-related event. The analysis showed that adherence levels were mostly low. We found that a factor that could be associated with low levels of adherence was level of education which in turn is related to inadequate health literacy. Initiatives to raise awareness of the issue are needed as well as dissemination of the fundamental principles underlying control strategies of nonadherence to therapy. Performing such initiatives requires a* collective*, joint effort that involves different categories of stakeholders, from patient to health care providers and policy makers, whose role may be different depending upon the kind of initiative and health system organization.

## Supplementary Material

Table S1: Shows the questionnaire administered to participants during Health Campus. The first part of the questionnaire has the purpose to collect demographic characteristics, socioeconomic and clinical information. The second part of the questionnaire report the MMAS-8 items.

## Figures and Tables

**Table 1:** Univariate association between low adherence on the Morisky scale and patient-reported factors.

	Low adherence	Medium adherence	Total	*P* value
	*N* = 187 (59.9%)	*N* = 125 (40.1%)	*N* = 312 (100.0%)	
Age group				0.58
Mean (±SD)	62.0 (11.4)	61.7 (10.8)	61.8 (11.1)	
18–60	75 (58.1)	54 (41.9)	129 (100.0)	
≥60	112 (61.2)	71 (38.8)	183 (100.0)	
Gender				0.03
Male	89 (66.9)	44 (33.1)	133 (100.0)	
Female	98 (54.7)	81 (45.3)	179 (100.0)	
Educational level				0.01
Primary school graduation	34 (68.0)	16 (32.0)	50 (100.0)	
Secondary school graduation	65 (69.9)	28 (30.1)	93 (100.0)	
High school graduation	55 (50.0)	55 (50.0)	110 (100.0)	
Degree graduation	33 (55.9)	26 (44.1)	59 (100.0)	
Number of medications				0.62
Mean (±SD)	2.33 (1.6)	2.15 (1.5)	2.26 (1.58)	
1	77 (56.6)	59 (43.4)	136 (100.0)	
2-3	71 (60.7)	46 (39.3)	117 (100.0)	
4-5	21 (67.7)	10 (32.3)	31 (100.0)	
≥6	18 (64.3)	10 (35.7)	28 (100.0)	
Smoking				0.03
No	136 (56.7)	104 (43.3)	240 (100.0)	
Yes	51 (70.8)	21 (29.2)	72 (100.0)	
Number of diseases				0.08
Mean (±SD)	1.58 (0.87)	1.46 (0.64)	1.53 (0.78)	
1	111 (59.4)	76 (40.6)	187 (100.0)	
2	52 (55.9)	41 (44.1)	93 (100.0)	
≥3	24 (75)	8 (25)	32 (100.0)	
Heart failure				0.58
No	171 (60.4)	112 (39.6)	283 (100.0)	
Yes	16 (55.2)	13 (44.8)	29 (100.0)	
COPD				0.35
No	183 (59.6)	124 (40.4)	307 (100.0)	
Yes	4 (80.0)	1 (20.0)	5 (100.0)	
Gastritis				0.42
No	172 (59.3)	118 (40.7)	290 (100.0)	
Yes	15 (68.2)	7 (31.8)	22 (100.0)	
Renal failure				0.68
No	185 (60.1)	123 (39.9)	308 (100.0)	
Yes	2 (50.0)	2 (50.0)	4 (100.0)	
Osteoporosis				0.15
No	168 (58.7)	118 (41.3)	286 (100.0)	
Yes	19 (73.1)	7 (26.9)	26 (100.0)	
Diabetes mellitus				0.51
No	163 (59.3)	112 (40.7)	275 (100.0)	
Yes	24 (64.9)	13 (35.1)	37 (100.0)	
Hypertension				0.76
No	87 (60.8)	56 (39.2)	143 (100.0)	
Yes	100 (59.2)	69 (40.8)	169 (100.0)	

**Table 2:** Association between adherence score with educational level and other significant variables in the regression analysis.

Variables	Odds ratio	IC 95%	*P* value
Gender			
Female	Reference		
Male	0.65	0.39–1.06	0.08
Smoker	Reference		
Nonsmoker	1.87	1.04–3.35	0.03
Educational level			
Primary school graduation	Reference		
Secondary school graduation	1.01	0.47–2.17	0.98
High school graduation	2.21	1.08–4.52	0.03
Degree graduation	1.63	0.74–3.62	0.23
