# Supplementary material for: Self-Assessment of Adherence to Medication: A Case Study in Campania Region Community-Dwelling Population
Source: J Aging Res. 2015 Aug 5;2015:682503. doi: 10.1155/2015/682503 (PMC4541012; doi:10.1155/2015/682503)
Supplement: Supplementary file 1 — Table S1: Shows the questionnaire administered to participants during Health Campus. The first part of the questionnaire has the purpose to collect demographic characteristics, socioeconomic and clinical information. The second part of the questionnaire report the MMAS-8 items. [file 682503.f1.pdf]

Table S1. Questionnaire used for interview including MMAS-8.

**Genaral information:**<br>Surname: ____________________ Name: ____________________<br>Gender: ☐ M ☐ F Age: ________			
**Education Level:**<br>☐ Primary school graduation ☐ Secondary school graduation<br>☐ High school graduation ☐ Degree graduation			
**Smoker:** ☐ Yes ☐ No			
**Diseases:**<br>☐ Hypertension ☐ Health failure ☐ COPD ☐ Diabetes<br>☐ Gastritis ☐ Renal failure ☐ Osteoporosis			
**N. of medications:** ☐ 1 ☐ 2 ☐ 3 ☐ 4 ☐ 5 ☐ 6 or more			
**©Morisky Medication Adherence Scale (MMAS-8-Item).**		**No**	**Yes**
1	Do you sometimes forget to take your medication(s)?		
2	People sometimes miss taking their medications for reasons other than forgetting. Thinking over the past two weeks, were there any days when you did not take your medication(s)?		
3	Have you ever cut back or stopped taking your medication(s) without telling your doctor, because you felt worse when you took it?		
4	When you travel or leave home, do you sometimes forget to bring along your medication(s)?		
5	Did you take your medication(s) yesterday?		
6	When you feel like your is under control, do you sometimes stop taking your medication(s)?		
7	Taking medication(s) everyday is a real inconvenience for some people. Do you ever feel hassled about sticking to your treatment plan?		
8	How often do you have difficulty remembering to take all your medication(s)?<br>☐ Never/Rarely ☐ Once in a while ☐ Sometimes ☐ Usually ☐ All the time		

Use of the ©MMAS is protected by US copyright laws. Permission for use is required. A license agreement is available from Donald E. Morisky, ScD, ScM, MSPH, Professor, Department of Community Health Sciences, UCLA School of Public Health, 650 Charles E. Young Drive South, Los Angeles, CA 90095-1772, dmorisky@ucla.edu
